# Association of hypoxia inducible factor-1 alpha gene polymorphism with both type 1 and type 2 diabetes in a Caucasian (Hungarian) sample

**DOI:** 10.1186/1471-2350-10-79

**Published:** 2009-08-19

**Authors:** Geza Nagy, Reka Kovacs-Nagy, Eva Kereszturi, Aniko Somogyi, Anna Szekely, Nora Nemeth, Nora Hosszufalusi, Pal Panczel, Zsolt Ronai, Maria Sasvari-Szekely

**Affiliations:** 1 2nd Department of Internal Medicine, Semmelweis University, H-1444 Budapest, POB 260, Hungary; 2 Department of Medical Chemistry, Molecular Biology and Pathobiochemistry, Semmelweis University, Budapest, Hungary; 3 Department of Psychology, Eötvös Loránd University, Budapest, Hungary; 4 3rd Department of Internal Medicine, Semmelweis University, Budapest, Hungary

## Abstract

**Background:**

Hypoxia inducible factor-1 alpha (HIF-1α) is a transcription factor that plays an important role in neo-vascularisation, embryonic pancreas beta-cell mass development, and beta cell protection. Recently a non synonymous single nucleotide polymorphism (g.C45035T SNP, rs11549465) of HIF-1α gene, resulting in the p.P582S amino acid change has been shown to be associated with type 2 diabetes (T2DM) in a Japanese population. Our aim was to replicate these findings on a Caucasian (Hungarian) population, as well as to study whether this genetic effect is restricted to T2DM or can be expanded to diabetes in general.

**Methods:**

A large Caucasian sample (N = 890) was recruited including 370 T2DM, 166 T1DM and 354 healthy subjects. Genotyping was validated by two independent methods: a restriction fragment analysis (RFLP) and a real time PCR using TaqMan probes. An overestimation of heterozygotes by RFLP was observed as a consequence of a nearby SNP (rs34005929). Therefore genotyping results of the justified TaqMan system were accepted. The measured genotype distribution corresponded to Hardy-Weinberg equilibrium (P = 0.740)

**Results:**

As the TT genotype was extremely rare in the population (0.6% in clinical sample and 2.5% in controls), the genotypes were grouped as T absent (CC) and T present (CT and TT). Genotype-wise analysis showed a significant increase of T present group in controls (24.0%) as compared to patients (16.8%, P = 0.008). This genetic effect was demonstrated in the separated samples of type 1 (15.1%, P = 0.020), and also in type 2 (17.6%, P = 0.032) diabetes. Allele-wise analysis gave identical results showing a higher frequency of the T allele in the control sample (13.3%) than in the clinical sample (8.7%, P = 0.002) with similar results in type 1 (7.8%, P = 0.010) and type 2 (9.1%, P = 0.011) diabetes. The odds ratio for diabetes (either type 1 or 2) was 1.56 in the presence of the C allele.

**Conclusion:**

We confirmed the protective effect of a rare genetic variant of HIF-1α gene against type 2 diabetes in a Caucasian sample. Moreover we demonstrated a genetic contribution of the same polymorphism in type 1 diabetes as well, supporting a possible overlap in pathomechanism for T2DM and a T1DM.

## Background

Type 1 (T1DM) and type 2 diabetes (T2DM) both result from the metabolic consequences of insufficient insulin effect, and have similar complications but appear to be due to completely distinct pathogenetic mechanisms. T1DM results from autoimmune β-cell destruction leading to insulin deficiency, whereas T2DM is the end point of a progressive insulin secretory defect on a background of insulin resistance [1].

The genetic background of both types of diabetes is undeniable and is being widely investigated. Both common forms are considered to be complex diseases caused by multiple environmental and genetic risk factors [2,3]. Previously genes such as the PPARγ, Kir6.2, Calpain-10, TCF7L2 and class 1 HLA genes, the CTLA-4, INS, PTPN22 have been proved to be related to the manifestation of T2 and T1DM respectively [4,5]. Due to previously observed differences in the clinical and pathogenic properties of T1 and T2DM, candidate gene studies rarely consider T2 candidate genes as potential T1 candidate genes and vice versa.

Despite the clear differences between T1 and T2DM there are indications of common etiological factors contributing to their manifestation. Investigations focusing on the pathogenesis of β cell dysfunction in T2DM have uncovered factors classically associated with T1DM. Latent autoimmune diabetes of adults (LADA) initially has a clinical presentation similar to T2DM and may comprise up to 15% of the patients originally diagnosed as having T2DM [6,7]. These patients, however, like in T1DM, exhibit anti β cell autoimmunity and develop a progressive β cell failure suggesting an overlap in etiology [8]. It is hypothesized that an accelerated β cell loss may be the link between T1DM and T2DM as post mortem examinations of human pancreatic tissue suggest that increased apoptosis is responsible for decreased pancreatic β cell mass in T2DM [9]. This notion is supported by *in vitro *and in vivo animal experiments, revealing apoptosis in incubated rodent β cells [10] and also in cultured human islets incubated in glucose concentrations similar to those seen in plasma of patients with T2DM [11], and also in the desert gerbil, during the progression to hyperglycaemia induced by a high-energy diet [12].

This growing evidence suggests that classification of diabetes into two distinct diseases may not reflect the true nature of the disorder [13]. Clearly, β cell dysfunction is a hallmark of both types of diabetes and a genetic variant that predisposes an individual toward reduced insulin secretion potentially could increase the risk of developing diabetes [14].

Hypoxia inducible factor 1 (HIF-1) is a key early mediator of the response to ischemia. The heterodimer of HIF-1α and β subunits is a potent transcription factor that promotes cell survival, glycolysis, and angiogenesis [15]. The transcriptional activity is primarily controlled by the oxygen-regulated breakdown of the α subunit [16]. The HIF-1α protein contains five functional domains. The basic helix-loop-helix (bHLH) domain is specifically required for the binding of DNA [17], the Per/Arnt/Sim (PAS) domain is involved in dimerisation of the α and β subunits. Transcriptional activation and interaction with coactivators are mediated by two transactivation domains in the C terminal half of HIF-1α, termed as N-terminal (N-TAD) and C-terminal (C-TAD) transactivation domains [18]. Negative regulation of HIF-1α under normoxic conditions occurs via the oxygen-dependent degradation (ODD) domain, which partly overlaps with N-TAD.[19,20] Under normoxic conditions, HIF-1α is hydroxylated on proline residues (P402, P564) by a family of oxygen-dependant prolyl hydroxylases which mediate high affinity binding to the von-Hippel-Landau (VHL) protein, a component of the E3 ubiquitin-protein ligase complex that ubiquitinates HIF-1α, thereby targeting it for degradation[21].

The HIF-1α gene is located at chromosome 14q21-q24, where the susceptibility locus to T2DM was localized in Finns [22]. In a recent study Yamada and colleagues [23] examined all regions of the HIF-1α gene in a Japanese population and found 32 SNPs, two of them located in exons. They found a significant association between T2DM and a non synonymous SNP in exone 12 (rs11549465) causing a change of proline to serine (p.P582S) in the expressed protein. Our aim was to replicate Yamada's study in a diabetic sample with a different ethnic origin, and investigate whether this genetic variant in the HIF-1α gene, as a shared genetic contributor plays a role in the development of not only T2DM but T1DM as well.

## Methods

### Patients

Diabetic patients were recruited randomly from the inpatient and outpatient services of the 2^nd ^and 3^rd ^Department of Internal Medicine at the Semmelweis University. The diagnosis of DM was based on fasting plasma glucose levels or 75 g oral glucose tolerance test according to the criteria of the WHO. The study was approved by the Local Ethics Committee (TUKEB). Every patient provided written informed consent for their participation.

The demographic variables of the samples were as follows. T1DM sample N = 166; age: 36.9 ± 13.1; 54.8% male and 45.2% female. T2DM sample N = 370, age: 64.0 ± 12.0; 41.9% male and 58.1% female. Control subjects without DM history were recruited at the Institute of Psychology, Eötvös Loránd University, N = 354; age: 25.1 ± 8.5; 32.5% male and 67.5% female. Both the clinical and the control samples were ethnically homogenous, of Caucasian origin, and consisted of unrelated individuals.

### Genotyping

Non-invasive DNA sampling was applied as described elsewhere [24]. DNA was isolated from buccal cells using the DNA-purification kit obtained from Gentra (Minneapolis, US).

#### Restriction fragment length polymorphism

A PCR-RFLP technique developed by Percy et al. [25] was applied with small modifications. Amplification was carried out using 1 μM of each primer (forward: 5' GTG TGG CCA TTG TAA AA 3', reverse: 5' AAC ACG TTA GGG CTT CTT 3'), 1× buffer and Q-solution (Qiagen), 200 μM dATP, dCTP, dGTP and dTTP, 0.1 U HotStarTaq DNA polymerase and approximately 5 ng genomic DNA in a final volume of 10 μL. Thermocycle: 95°C for 15 minutes, followed by 40 cycles of 94°C, 30 sec denaturation, 52°C, 30 sec annealing and 72°C 1 min extension, with the final polymerization at 72°C for 10 minutes.

For the genotype dependent digestion 1× NEBuffer 1, 0.1 mg/mL BSA, 0.7 U Tsp45 I restriction endonuclease (New England Biolabs) and 5 μL PCR-product were used in a total volume of 50 μL. Samples were incubated at 65°C overnight and the generated DNA fragments were separated on 1.5% agarose-2% Metaphor agarose composite gel matrix. For controlling the complete digestion, a non-polymorphic cleavage site was applied producing a 194-bp-long fragment. A 260-bp-long product was observed in case of the T allele, while a 139 bp and 121 bp fragments were generated in the presence of the C variant.

#### Real-time PCR

The C__25473074_10 SNP genotyping kit obtained from Applied Biosystems contained the two flanking primers and the C- and T-specific probes labeled with VIC and FAM fluorescent dyes, respectively. 1× of this genotyping kit, 1× TaqMan^® ^PCR Master Mix, No AmpErase^® ^UNG and approximately 5 ng genomic DNA was employed in a final volume of 5 μL. A 7300 Real-Time PCR System was used for the amplification, the first step of the thermocycle was an initial denaturation and activation at 95°C for 10 minutes, followed by 40 cycles of 95°C for 15 seconds and 60°C for 1 minute.

### Comparison of the genotyping methods

150 healthy individuals were investigated using the two independent techniques. In more than 10% of the heterozygotes the *Tsp*45 I PCR-RFLP and the TaqMan method gave controversial results. Direct sequencing of the PCR products clearly demonstrated that there was a misgenotyping of rs11549465 SNP by PCR-RFLP. The misgenotyping was fully explained by the presence of another SNP (rs34005929) located in a 4 bp distance thus disturbing the correct genotyping of the studied polymorphism.

The allele discrimination of TaqMan system proved to be reliable based on direct sequencing data. Genotypes could be unambiguously determined on the observed *ΔC*_T _(*C*_T VIC _- *C*_T FAM_) values. *ΔC*_T _values between -5.0 and -2.4 corresponded to the CC genotype, values from -1.9 to 0.7 demonstrated the heterozygote form, whereas *ΔC*_T_-s in the range of 1.2–3.8 were characteristic to the TT variation. Therefore the TaqMan system was used for large scale genotyping. Obtained genotype frequencies were compared to the calculated frequencies based on the Hardy-Weinberg equilibrium and no significant differences were observed (P = 0.740 for all participants, P = 0.444 for the control group, P = 0.853 for the total patient group. Within the patient group P = 1.000 for the DM1 and P = 0.808 for the DM2 groups).

### Plasmid constructs

**Figure 1:**
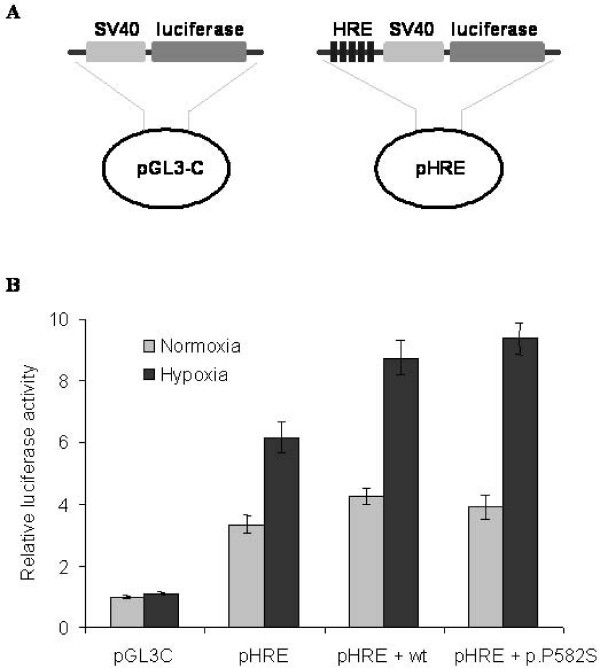
**The effect of the p.P582S mutation on the binding affinity of the HIF-1α to hypoxia responsive element (HRE) in SK-N-FI cells**. **A**. Schematic description of pHRE vector, which is a modified pGL3-Control plasmid, containing five HIF-1α binding sites (HRE) in front of the SV40 promoter and the luciferase reporter gene. **B**. No significant difference could be detected between the transcriptional activities of the pHRE constructs co-transfected with either wild type or p.P582S mutant HIF-1α, neither in normoxic nor under hypoxic conditions. Luciferase activity was normalized to the *β*-galactosidase activity. Data are presented as fold increments over the normoxic pGL3-Control activity and shown as mean ± SD. Results of a representative experiment are shown as measured in triplicates. Similar data were obtained from three independent transfection experiments.

The pGL3-Control luciferase reporter vector (Promega, Madison, WI) was used as a control. The pHRE vector – which is a modified pGL3-Control plasmid (figure 1A) – containing five contiguous hypoxia responsive elements (5'-GATCTGAGACAGCACGTAGGGC-3') in front of the luciferase reporter gene, was a generous gift from Dr. M. Geiszt (Institute of Physiology, Semmelweis University, Budapest, Hungary). The wild type and p.P582S mutant HIF-1α expression vectors were a kind gift of Dr. Yukio Horikawa, Department of Diabetes and Endocrinology, Gifu University School of Medicine, Gifu, Japan.

### Cell cultures, hypoxic treatment, transient transfection

SK-N-FI (neuroblastoma) cell line was grown in Dulbecco's modified Eagle's medium, high Glucose (Gibco, Carlsbad, CA) supplemented with 10% fetal bovine serum and 1% nonessential amino acids. Normoxic cultures were kept in 21% O_2_, 74% N_2 _and 5% CO_2 _in humidified atmosphere. Hypoxic samples were incubated in a humidified atmosphere of 1% O_2_, 94% N_2 _and 5% CO_2 _in a modular incubator chamber (Billups-Rothenberg, USA). A mixture of 0.1 μg pHRE reporter construct, 0.1 μg HIF-1α expression vector and 0.1 μg pCMV-*β*-gal and 6 μl Lipofectamine 2000 (Invitrogen, Carlsbad, CA) was used to transfect 1.2 × 10^6 ^SK-N-FI cells plated 24 hours before transfection in six-well plates. Luciferase and *β*-galactosidase activities were detected using the Luciferase Assay System kit (Promega, Madison, WI) and by ONPG (O-nitrophenyl-*β*-D-galactopyranoside) cleavage rate, respectively. Three parallels were used in all transfections and all experiments were performed in triplicates.

### Statistical analyses

The SPSS program for Windows (13.0 version) was used for the statistical analyses. The Hardy-Weinberg equilibrium and differences in allele and genotype distribution of controls and patient groups (or males and females) was tested by Chi-square analysis. Risks were examined by odds ratios (ORs) with 95% confidence intervals (CIs), using unconditional logistic regression models adjusted for age (years) and sex. Possible effects of genotypes on age or BMI were tested by t-tests. G*Power 3.1.0 [26] has been used for computing a post hoc test for achieved power. Input parameters included the population effect size = 0.1 (as a conventionally small effect size), alpha = 0.05, df = 1 for our 2 × 2 and df = 2 for our 2 × 3 analyses. Power estimates were 85% for the 2 × 2 contingency tables and 77% for the 2 × 3 contingency tables, which are generally considered acceptable [27].

## Results

DNA samples of 536 patients and 354 controls were genotyped for the rs11549465 of HIF-1α gene. Considering previous reports indicating that gender might alter glucose homeostasis and the development of diabetes [28,29] as a first step of analysis we excluded any possible significant effect of sex on genotype distribution in the various study groups. Table 1 shows the percentages of genotypes calculated for males and females, and the number of individuals in brackets. As the TT genotype was extremely rare in the population, they were grouped together with CT heterozygotes for this analysis. Using Pearson Chi-Square test we did not find any significant effect of sex on genotype distribution in Controls (P = 0.379), T1DM (P = 0.435) or in T2DM (P = 0.624) samples. Possible association of the HIF-1α genotypes and age or BMI index has also been tested by using the T-present vs. T-absent genotypes as independent groups. Analyses were performed independently in the control, DM1 and DM2 groups, (data on BMI index of controls was available for only 50 subjects). No significant associations were found, mean age and mean BMI of the tested T-present and T-absent genotype groups were similar (data not shown).

**Table 1:** Distribution of Hypoxia inducible factor-1 alpha (HIF-1α) genotypes (rs11549465) between males and females

	**Genotypes**	**Male**	**Female**
**Control**	CC	73.0% (84)	77.4% (185)
	CT + TT	27.0% (31)	22.6% (54)

**Type 1 Diabetes**	CC	86.8% (79)	82.7% (62)
	CT+TT	13.2% (12)	17.3% (13)

**Type 2 Diabetes**	CC	81.3% (126)	83.3% (179)
	CT+TT	18.7% (29)	16.7% (36)

The main goal of our analysis was to study the association between the HIF-1α gene polymorphism and diabetes. Table 2 presents the allele and genotype frequencies in the control and the patient group as a total ("all patients"), and in the separated groups of type 1 (T1DM) and type 2 (T2DM) diabetes. We found a statistically significant difference regarding both the genotype and allele distribution between patients and controls. Genotype-wise analysis demonstrated a significant increase of CC homozygotes among patients (83.2%) compared to the controls (76%), *χ*^2^(2) = 10,792, P = 0.005. In accordance with this, the allele-wise analysis verified a lower frequency of T allele among patients (8.7%) than controls (13.3%), *χ*^2^(1) = 9,602, P = 0.002.

**Table 2:** Hypoxia inducible factor-1 alpha (HIF-1α) allele and genotype frequencies in the studied groups

**Genotypes**	**CC**	**CT**	**TT**	
**Control**	76.0% (269)	21.5% (76)	2.5% (9)	
**All patients**	83.2% (446)	16.2% (87)	0.6% (3)	**P = 0.005**
**T1DM**	84.9% (141)	14.5% (24)	0.6% (1)	P = 0.044
**T2DM**	82.4% (305)	17.0% (63)	0.6% (2)	P = 0.023

**Grouped genotypes**	**CC**	**CT + TT**		

**Control**	76.0% (269)	24.0% (85)		
**All patients**	83.2% (446)	16.8% (90)		**P = 0.008**
**T1DM**	84.9% (141)	15.1% (25)		**P = 0.020**
**T2DM**	82.4% (305)	17.6% (65)		**P = 0.032**

**Alleles**	**C**	**T**		

**Control**	86.7% (614)	13.3% (94)		
**All patients**	91.3% (979)	8.7% (93)		**P = 0.002**
**T1DM**	92.2% (306)	7.8% (26)		**P = 0.010**
**T2DM**	90.9% (673)	9.1% (67)		**P = 0.011**

Separation of diabetic patients to type 1 (T1DM) and type 2 (T2DM) gave identical results demonstrating that the effects of HIF-1α gene variants are similar in type 1 and type 2 diabetes (Table 2). As the T allele is relatively rare especially among patients (8.7%), TT homozygotes do not reach the criteria of Pearson Chi-Square test in some cases (P values are in parentheses in Table 2). Therefore it was necessary to group the genotype categories as T absent (CC) and T present (CT and TT) for genotype-wise statistical analysis. As it is shown in Table 2, the allele and genotype distributions between type 1 and type 2 patients are approximately equal and both groups show a statistically significant difference if compared to the control group. The values of significance (P) are given in Table 2 regarding the comparison between the patients and the controls. It is important to note that comparison of either allele or genotype distribution among patients with type 1 and type 2 diabetes did not result in any significant differences.

The presented data demonstrated an under representation of the T allele in all patient groups suggesting a protective effect of the T allele against diabetes. It is worth mentioning that -although the numbers are rather small – TT homozygotes are extremely rare among patients (0.6%), while their incidence is more than 4 fold higher in the control group (2.5%). With other words, the C allele is a risk factor for both type 1 and type 2 diabetes (OR = 1.56, CI: 1.41–1.71). The above risk estimate was based on unconditional logistic regression adjusted for age (years) and sex using data from a total of 879 participants with all data available (535 patients and 344 controls).

In order to gain more evidence for the functional role of P582S amino acid change, transcriptional activity of the allelic variants were compared in an *in vitro *reporter gene system (see Figure 1). Five contiguous hypoxia responsive elements (HRE) were inserted into the pGL3-Control plasmid (pGL3-C) for the assay of HIF-1α transcriptional activity (see Figure 1A). Two allelic forms (wt = wild type, p.P582S = rare variant) of HIF-1α expression vector were co-transfected with pHRE in separate experiments using SK-N-FI (neuroblastoma) cell line. Relative HIF-1α activity was measured in cell extracts as a ratio of luciferase activity and the *β*-galactosidase activity applied as transfection control. The obtained results were normalized for the low activity of "empty" vector (pGL3-C) under normoxia, and labeled on Figure 1. as relative luciferase activity. As expected, a 2–3 fold HIF-1α transcriptional activity was measured under hypoxia (filled columns) compared to normoxia (open columns), except in case of "empty" vector. No significant differences were found between allelic variants (pHRE + wt and pHRE + p.P582S) either under normoxia or under hypoxia in our conditions. It should be noted, however, that the endogenous HIF-1α activity was relatively high in our system, as measured by pHRE without cotransfection of any of the allelic variants.

## Discussion

In the present genetic association analysis performed on a Caucasian sample, we investigated the HIF-1α gene rs11549465 SNP, a C→T non synonymous SNP (g.C45035T) resulting in a substitution of proline to serine (P582S) in exon 12. Our result demonstrated a statistically significant decrease in frequencies of T allele containing genotypes (CT + TT), as well as in the T allele frequencies among individuals with diabetes (see Table 2). Of note, allele and genotype distribution was very similar between subjects with T1DM and those with T2DM. On the other hand both the T2DM and T1DM groups showed a statistically significant difference in allele and genotype distribution when compared to the control. In summary, a protective effect of the rare HIF-1α gene variant was proven against both, type 1 and type 2 diabetes, in a Caucasian sample.

As it was pointed out by earlier studies, it is highly relevant to replicate genetic association studies, because it is well known that there are significant differences in the frequencies of certain genetic variations among different ethnic groups. Our results regarding T2DM patients are in line with the first demonstration of association between the T allele in exon 12 of the HIF-1α gene and T2DM by Yamada and his colleagues. The frequency of the rare T allele in our control sample (13.3%) was comparable to previous reports on the European population [30], and indicated that the T allele is almost twice as frequent among Caucasians, than observed in Yamada's Japanese study group.

Due to the substantially different pathophysiological features of T1 and T2DM, candidate genes of T2DM have rarely been examined as candidate genes modifying the risk of T1DM [31,32]. However recently an overlap has been proposed by Wilkin and coworkers [33]. Their "accelerator hypothesis" suggests that T1 and T2 diabetes are the same disease of hyperglycemia-induced beta cell damage in which T1DM has the added effect of autoimmunity. Other findings have also strengthened this hypothesis from a genetic point of view by demonstrating familiar clustering of type 1 and type 2 diabetes mellitus [34-36]. There is also evidence that for selected susceptibility gene variants, there might be a shared genetic contribution to the pathogenesis of T1DM and T2DM. For example, the common variant of the peroxisome proliferator activated receptor γ gene isoform 2 (PPARγ2) Pro12Ala that has been consistently reported to associate with T2DM was recently shown to be associated with T1DM, as well [37]. Moreover, Galanakis and coworkers just recently have shown that the intron 4 a/b polymorphism of the endothelial nitric oxide synthase gene (eNOS) is associated with both type 1 and type 2 diabetes [38]. Our new finding that indicates a decreased number of the minor allele in the T1DM sample is another example for the possible shared genetic background of T2 and T1 diabetes.

The polymorphism investigated in our study causes a proline to serine change in the 582 position which is within the N-TAD near the ODD domain of the HIF-1α protein [39]. Proline 582 has not been proven to be a HIF-1α hydroxylation site, and it is not known whether it mediates VHL binding. Moreover, the serine-proline substitution in this position does not appear to alter VHL binding *in vitro *to a fragment of HIF-1α after hydroxylation at proline 564 [25].

Previous *in vitro *functional analysis of this HIF-1α mutation gave conflicting results. First Yamada and his colleagues [23] indicated that the mutant variant has a consistently higher level of HIF-1α transcriptional activity than the wild-type. Since the enhanced transactivation capacity of the mutant was observed with statistical significance only under hypoxic condition, the authors suggested that this genetic variant, by enhancing the transcriptional activity of target genes, could be a protective factor against the onset of type 2 diabetes by its activities in the pancreatic developmental stage. Hlatky and coworkers [30] could not replicate these findings. We also attempted to demonstrate the functional importance of HIF-1α variants, however, we did not find any significant differences in the transcriptional activity of the HIF-1α variants using a luciferase reporter system (see Figure 1). One possible reason of this contradiction might be a relatively high endogenous HIF-1α activity in our cell line as measured in the presence of the luciferase vector with hypoxia responsive elements (Figure 1, pHRE) in the absence of any HIF-1α expression vector.

Tanimoto and colleagues suggested that the conformational changes caused by the amino acid substitution either might alter protein stability, or could enhance recruitment of transcriptional cofactors that interact with HIF-1α. Since, the authors could not detect any differences in degradation, the altered transactivational properties was taken into consideration as a possible molecular effect of Pro582Ser change [40]. Further investigations in the field of cancer research demonstrated the rs11549465 variant to have enhanced transcription activities in *in-vitro *studies under both normoxic and hypoxic conditions [40,41] associated with increased tumor microvessel density in head and neck cancer, and in prostate cancer. In conclusion, changes in the transactivational properties of the studied genetic variants could be hypothesized, however, their effect probably depends on the specific coactivators of various cell types.

HIF-1 is a major determinant in the expression and secretion of vascular endothelial growth factor (VEGF) by cells [42-44]. It has been shown that VEGF increases the survival of pancreatic islets and thus β cell sparing after islet transplantation by stimulating angiogenesis and improving islet revascularization [45,46]. Moreover, transgenic mice that over-express VEGF are characterized by islet hyperplasia, suggesting that VEGF modulates endocrine pancreatic differentiation [47]. Recent findings suggest that VEGF protects also against the development of T1DM and may play a role as a specific "pancreatic protector" [48]. Therefore, one might speculate that the studied genetic variant of the HIF-1α, by enhancing the transcriptional activity of target genes, could be a protective factor against the onset of not only type 2 diabetes [23] but also that of type 1 diabetes by its activities in the pancreatic developmental stage.

## Conclusion

In the presented genetic association study we determined the genotype of the HIF-1α gene rs11549465 SNP, a C→T non synonymous SNP (g.C45035T) resulting in a substitution of proline to serine (P582S) in exon 12 on a large Caucasian sample of diabetic patients (N = 536) and controls (N = 354). In accordance with a recent study of Yamada and colleagues [23], we found a statistically significant decrease in frequencies of CT and TT genotypes, as well as in the T allele frequencies among individuals with diabetes suggesting the protective effect of the T allele in type 2 diabetes. Moreover, the same genetic effect was found in type 1 diabetes, as well, pinpointing the possibility of shared genetic contributors in the development of diabetes mellitus.

## Competing interests

The authors declare that they have no competing interests.

## Authors' contributions

GN conducted the clinical study by the direction of AS. RKN carried out the molecular genetic studies, NN developed the genotyping methodologies. EK performed the functional study using reporter gene constructs. Design and evaluation of the molecular genetic studies was made by ZR. ASZ performed the statistical analysis. NH participated in collection of type 1, PP was responsible for type 1 samples. MSSZ conceived of the study, and participated in its design and coordination and helped to draft the manuscript. All authors read and approved the final manuscript.

## Pre-publication history

The pre-publication history for this paper can be accessed here:


